# Green-Synthesis of Anisotropic Peptone-Silver Nanoparticles and Its Potential Application as Anti-Bacterial Agent

**DOI:** 10.3390/polym11020271

**Published:** 2019-02-05

**Authors:** Min Kim, Seung-Cheol Jee, Surendra K. Shinde, Bhupendra M. Mistry, Rijuta Ganesh Saratale, Ganesh Dattatraya Saratale, Gajanan S. Ghodake, Dae-Young Kim, Jung-Suk Sung, Avinash A. Kadam

**Affiliations:** 1 Department of Life Sciences, Dongguk University-Seoul, Biomedi Campus, 32 Dongguk-ro, Ilsandong-gu, Goyang-si 10326, Gyeonggi-do, Korea; pipikimmin@naver.com (M.K.); markjee@naver.com (S.-C.J.); sungjs@dongguk.edu (J.-S.S.); 2 Department of Biological and Environmental Science, Dongguk University-Seoul, Biomedical Campus, Ilsandong-gu, Goyang-si 10326, Gyeonggi-do, Korea; surendrashinde.phy@gmail.com (S.K.S.); ghodakegs@gmail.com (G.S.G.); sbpkim@dongguk.edu (D.-Y.K.); 3 Department of Food Science and Biotechnology, Dongguk University-Seoul, Ilsandong-gu, Goyang-si 10326, Gyeonggi-do, Korea; b.mistry84@dongguk.edu (B.M.M.); gdsaratale@gmail.com (G.D.S.); 4 Research Institute of Biotechnology and Medical Converged Science, Dongguk University-Seoul, Biomedi Campus, Ilsandong-gu, Goyang-si 10326, Gyeonggi-do, Korea; rijutaganesh@gmail.com

**Keywords:** Peptone, Microbial nutrient, Anti-bacterial silver nanoparticle, *Escherichia coli*, *Staphylococcus aureus*

## Abstract

This study demonstrates a green-route-based synthesis of high-concentration suspensions of anisotropic silver nanoparticles (AgNPs) by peptone (Pep), a soluble protein hydrolysate and an abundantly used nutrient source in microbial-media. The transformation of Ag ions from solution into a high-concentration suspension of anisotropic Pep-AgNPs, at an extremely low concentration of peptone (0.02%), indicates that the present green-route synthesis method follows “low volume high concentration nano-synthesis”, and, hence, enhances the economic significance of the process. Process optimization with different concentrations of AgNPs (1–5 mM), NaOH solution (5–40 mM), and peptone (0.004%–0.12%) gave the optimized Pep-AgNPs synthesis at 3 mM of AgNO_3_, 20 mM of NaOH, and 0.02% of the peptone concentrations. The green-route synthesized Pep-AgNPs were structurally characterized by the TEM, XPS, FT-IR, and XRD analyses. The Pep-AgNPs against the clinically relevant bacteria *Escherichia coli* and *Staphylococcus aureus* gave significant anti-bacterial properties, with a MIC (minimum inhibitory concentration) of 100 ppm. The colony counting and morphological observation of the bacterial cell under SEM corroborated an anti-bacterial potential of the Pep-AgNPs. Therefore, Pep-AgNPs are green-route synthesized, anisotropic, and have a significant anti-bacterial potential that can be used in many relevant applications.

## 1. Introduction

As the development of nanotechnology progresses, the silver nanoparticles (AgNPs) have become one of the most demanding nanoparticles, owing to their increasing number of applications in different sectors [1,2,3,4,5,6,7,8,9]. The shape, surface chemistry, and size of the AgNPs gives them typical physical, optical, chemical, and electronic properties. Therefore, the specific design of AgNPs has been a central research topic for several years [7,10,11,12,13,14,15]. However, chemically synthesized AgNPs, using chemical agents such as hydrazine, sodium borohydride (NaBH_4_), and dimethylformamide, exert significant environmental cytotoxicity [16,17]. An alternative method, such as the whole cell-based AgNPs synthesis, also encompasses several limitations, such as adsorption of nanoparticles to the cell surface, downstream processing, and significantly less control over the shape and size [16]. Greener-route molecular manufacturing of AgNPs would be more economical, reproducible, and a more sustainable process [13,17,18,19]. The green-route synthesis of nanomaterials has significantly increased in the past few decades [20]. The greener nanoscience approach mainly aims to create and apply design rules proactively, and to develop efficient synthetic strategies to produce nano-materials with defined composition, structure, and purity [20]. Therefore, to fulfill the enormous demand in the growing nanotechnology market, rapid, high concentration, and greener-route synthesis hold significant importance.

Over time, there has been a continuous increase in the number of multidrug-resistant bacterial strains, due to pollution, mutation, and the changing environmental conditions [21]. To sidestep this difficulty, scientists are developing drugs for the treatment of these harder-to-treat microbial infections [21]. Numerous metal salts and metal oxide nanoparticles were applied to the task of inhibiting the growth of many infectious bacteria. Among these materials, AgNPs occupy a prominent place [5,14,16,17,20,22]. However, in the last decade, there has been a rapid upsurge in the greener-route synthesized AgNPs. Still, the fabrication of specifically targetable AgNPs is extremely needed in the present day to combat the upcoming challenges in microbial infections. 

Peptone (Pep), a component of microbiological media, is mainly defined as protein hydrolysates prepared from proteinaceous materials [23]. They are mainly utilized as an assimilable nitrogen source in microbial media [23]. The basic peptone composition contains mainly amino acids, short chain peptides, and proteins. Due to the presence of different components, such as amino acids, short chain peptides, and proteins, it might be possible to produce a mixture of different-shaped AgNPs possessing the antimicrobial potential. While investigating the detailed mechanism of AgNPs synthesis by microorganisms, role microbial media component peptone I in AgNPs synthesis was mentioned [24]. However, the peptone capping for AgNPs for detailed anti-microbial assessment was not reported elsewhere in the literature. Therefore, in this study, facile and rapid synthesis of peptone-capped AgNPs with optimization of process parameters, detailed characterizations, and antimicrobial assessment was investigated. The synthesis of AgNPs with the peptones might give several advanced antimicrobial applications. 

Therefore, this study aims at the rapid green-route synthesis of microbial nutrient peptone-coated silver nanoparticles (Pep-AgNPs) as an economically and environmentally feasible nano-formulation for accessing its potential in anti-bacterial applications. Pep-AgNPs were green-route facile synthesized, optimized for the process parameters, structurally characterized with TEM, FT-IR, XPS and XRD analyze, and finally applied for accessing the anti-bacterial potential against the clinically relevant *Escherichia coli* and *Staphylococcus aureus*. 

## 2. Materials and Methods

### 2.1. Green-Route Synthesis of Pep-AgNPs

The chemicals, such as BD Bacto™ peptone and silver nitrate, were obtained from BD Biosciences, San Jose, CA, USA, and Sigma Aldrich, St. Louis, MI, USA, respectively. A stock solution of peptone (0.4% wt/v) was prepared using DI (deionized) water and used freshly in the synthesis of Pep-AgNPs. Synthesis of Pep-AgNPs was examined by varying concentrations of precursor reagents and alkaline additive (NaOH). A solution of NaOH was obtained from Dae Jung Chemicals, Shiheung, Korea. The effect of the peptone amount was tested at increasing concentrations (0.0, 0.004, 0.012, 0.024, 0.040, 0.08, and 0.12% wt/v) with 0.2 mL of NaOH (1 M) and 0.2 mL of AgNO_3_ (20 mM) in 10 mL reaction mixture. The effect of NaOH concentration at 5, 10, 20, 30, and 40 mM was studied with a fixed amount of peptone (0.04% wt/v) and AgNO_3_ (0.4 mM) in 10 mL aqueous reaction mixture. The initial AgNO_3_ concentration varied from 1.0 to 5 mM, with a fixed amount of aqueous peptone (0.04% wt/v) and 0.2 mL of NaOH (1 M) in 10 mL aqueous reaction mixture. The AgNP reactions were run at 65 °C for 48 h incubation and used to record the UV-vis absorption spectrum on a UV-vis spectrophotometer (Optizen 2120, Mecasys Co., Ltd. Banseok-dong, Yuseong-gu, Daejeon, Korea). The centrifugation process was used to remove any unbound silver ions and peptone components, followed by the thorough washing of Pep-AgNPs with DI water. Typically, well-washed Pep-AgNPs solution was diluted accordingly and placed in a cell for spectral analysis in the range of 300–800 nm. The synthesized nanoparticles were stored at room temperature (22–24 °C).

### 2.2. Characterization of Pep-AgNPs

A Pep-AgNPs sample was drop-cast onto a piece of the Formvar-coated 200-mesh copper grid and was visualized using a transmission electron microscope (TEM) (Technai G2, Ames Laboratory, Ames, Iowa). The size of ~164 nanoparticles were measured to determine the size-distribution pattern. X-ray diffraction (XRD) spectrum in the 2θ range of 20°–80° was obtained on a diffractometer system (X’Pert PRO, PANalytical, Malvern, UK) operated at 40 kV, and 30 mA spectrum was recorded by Cu-Kα radiation with a wavelength of 1.5406 Å. The chemical characterization of the Pep-AgNPs thin film was performed by X-ray photoemission spectroscopy (XPS), and was operated with Theta Probe AR-XPS System, Thermo Fisher Scientific, Waltham, MA, USA, in the constant-pass energy mode with a monochromatic Al-Kα X-ray source. The binding energy scale of the XPS spectrum was calibrated by measuring the C1s peak at 285.0 eV. Fourier transform infrared (FTIR) spectrum of the centrifuged Pep-AgNPs was recorded over a spectral range of 500–3500 cm^−1^ to identify the functional groups (Thermo Fisher Scientific, Nicolet iS10, UK).

### 2.3. Microbial Strains

*Escherichia coli* (KCCM 11234; *E. coli*) and *Staphylococcus aureus* (KCCM 11335; *S. aureus*) were obtained from the Korean Culture Center of Microorganisms (KCCM, Seoul, Korea). Each bacterium was cultivated on a Tryptic Soy Agar plate (TSA; BD, San Jose, CA, USA) at 37 °C for overnight. A single colony of each bacterium was inoculated in Tryptic Soy Broth (TSB; BD, San Jose, CA, USA) and incubated at 37 °C 150 rpm for 18 h. To analyze the anti-bacterial effect of nanoparticles, bacterial cells which had the exponential phase OD600 = 0.4−0.6 were used. 

### 2.4. Antibacterial Test 

Antibacterial effects of nanoparticles were evaluated against gram-negative *E. coli* and gram-positive *S. aureus*. The bacterial suspension was diluted to 10^5^ CFU/mL with TSB and 100 μL was dispensed into each well of the 96-well microplate. Then, various concentrations of Pep-AgNPs were added into each well, which had inoculated bacteria. The inoculated 96-well microplates were incubated at 37 °C for 4 h. After incubation, Pep-AgNPs containing TSB were removed and replaced with Pep-AgNPs free media, prior to turbidity measurement. Its turbidity was measured under 600 nm of wavelength using an ELISA reader (Tecan, Männedorf, Switzerland). All data were analyzed statistically using one-way analysis of variance (one-way ANOVA). Minimal inhibitory concentration (MIC) was determined as the lowest concentration without bacterial growth. After measurement of turbidity, to observed colony formation assessment, 100 μL volumes from each well of the microplates were aspirated and diluted to 10^−5^. Dilutions were cultured on TSA for 16–18 h. Colony-forming units were manually counted.

### 2.5. SEM Analysis of Bacterial Surface Morphology Observation

To attach the bacterium cells on cover-slips, a cover-slip was coated with poly-L-lysine (Sigma, St. Louis, MI, USA) according to the manufacturers protocols. A bacterial suspension of 107 CFU/mL was added on a coverslip for 1 h. To evaluate the destruction of the bacterial cell wall, the cover-slip was washed with phosphate-buffered saline (PBS, Bio-sensing, Seoul, Korea), and nanoparticle suspension was treated on it for 2 h. After incubation, the bacterium was fixed with 2.5% glutaraldehyde for 24 h at 4 °C. For dehydration, 30, 50, 60, 70, 80, 90, and 100% of ethanol were gradually treated for 10 min each. 

## 3. Results and Discussions

### 3.1. Pep-AgNPs Synthesis 

**Figure 1:**
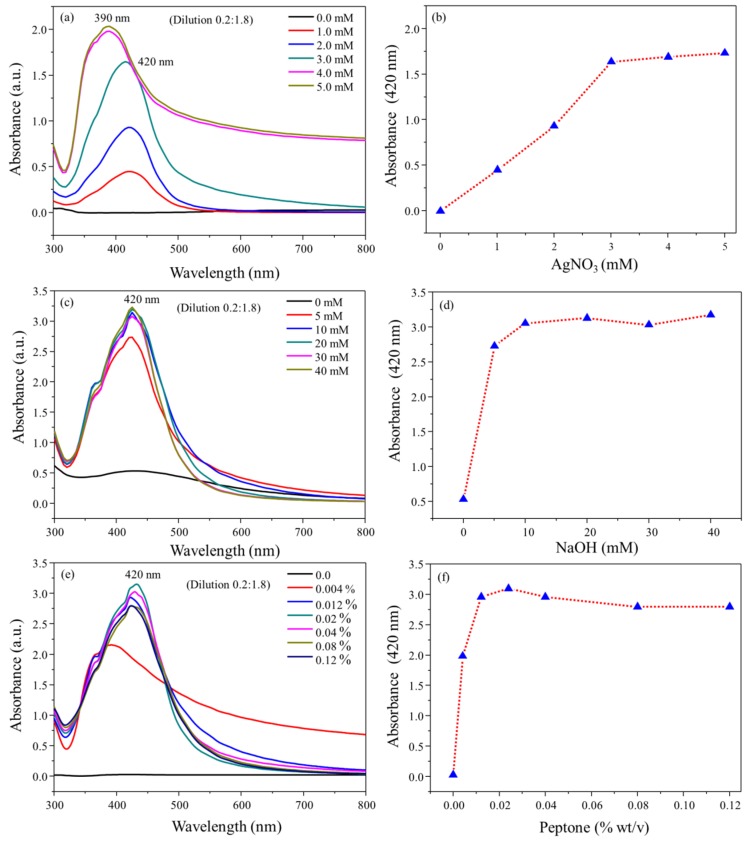
(**a**) Uv-vis wavelength scan of Pep-AgNPs synthesis at various AgNO_3_ concentrations, (**b**) Absorbance at 420 nm for Pep-AgNPs synthesis at various AgNO_3_ concentrations, (**c**) Uv-vis wavelength scan of Pep-AgNPs synthesis at various NaOH concentrations, (**d**) Absorbance at 420 nm for Pep-AgNPs synthesis at various NaOH concentrations, (**e**) Uv-vis wavelength scan of Pep-AgNPs synthesis at various peptone (Pep) concentrations and (**f**) Absorbance at 420 nm for Pep-AgNPs synthesis at various peptone concentrations.

This study was designed to produce AgNPs under a direct molecular reduction mechanism, using diluted peptone water as a strong reducing and stabilizing agent. Hence, the present process was totally based on green chemistry principles. The formation of Pep-AgNPs was elicited by the addition of the aqueous NaOH solution, mainly due to the catalytic properties of OH- and/or hydration of peptone functional groups. The environmentally benign solvent water was used as a solvent for all the studies, including the preparation and storage of Pep-AgNPs. In the present work, peptone-capped AgNPs (Pep-AgNPs) were synthesized at a very low concentration of peptone and a temperature of 65 °C. Uv-vis spectrum of Pep-AgNPs synthesized at various initial concentrations (1–5 mM) of AgNO_3_ with a dilution of (1:10) of synthesized nanoparticles were shown in Figure 1a. The obtained results clearly revealed significant enhancement in surface plasmon resonance of Pep-AgNPs, with a subsequent increase in the AgNO_3_ concentration. The obtained absorbance unit of 2.1 for (1:10 diluted) at 5 mM AgNO_3_ concentration corroborated a very high concentration synthesis of Pep-AgNPs. Figure 1b showed a subsequent increase in the concentrations of AgNO_3,_ which increased the concentration of Pep-AgNPs synthesis. The highest concentration of Pep-AgNPs was observed at the concentration of 3 mM, and remained stable through further increases in the concentrations. The optical properties of silver nanoparticles are highly reliant on the diameter of nanoparticles [24]. It is typically recognized that blue-shift of the plasmon band position occurs for a decrease in the particle size [24]. Hence, at the concentration of 4 and 5 mM of AgNO_3_, the blue shift was observed in surface plasmon resonance of the Pep-AgNPs, therefore, increased absorbance with blue shift corroborated a high concentration synthesis with the reduced particle size of Pep-AgNPs. In the next step, the effect of NaOH concentrations on Pep-AgNPs synthesis was investigated (Figure 1c). An increase in the concentration of NaOH showed an increase in the Pep-AgNPs synthesis. The optimum NaOH concentration was obtained to be 20 mM, and beyond this concentration, the *Pep-AgNPs* nanoparticles remained stable with no further increase (Figure 1d). To make a specific coating of proteinaceous materials from peptone on AgNPs, and to make the process more economical, it was highly important to access the effect of various concentrations of peptone for Pep-AgNPs synthesis. The effect of increasing peptone concentrations is displayed in Figure 1e. The obtained results corroborate the significant increase in the Pep-AgNPs synthesis with an increase in peptone concentration until the 0.024%, however, further increases in the peptone concentration Pep-AgNPs synthesis decreased with specific blue/red shift effect. At the concentration of 0.004%, surface plasmon resonance showed absorption maxima at 400 nm. However, from the peptone concentrations of 0.004% to 0.12%, it gave the absorption maxima at 420 nm, respectively. These results corroborated that increases in the peptone concentration above 0.004% changed the surface plasmon resonance properties of Pep-AgNPs. The highest synthesis was observed at the concentration of 0.02% of peptone. The almost complete transformation of Ag ions into an identical Pep-AgNPs, at an extremely low concentration of peptone (0.02%), indicates that the present green-route synthesis method is highly suitable for the “low volume high concentration nano-synthesis”, and enhances the economic significance. The overall optimizations of AgNO_3_, NaOH, and peptone solution concentrations for Pep-AgNPs synthesis corroborated a well-optimized synthesis process. The synthesized Pep-AgNPs were further used for characterizations and anti-bacterial studies. In comparison to other AgNPs reported, the synthesized Pep-AgNPs are coated with microbial nutrients, hence, the micro-organism might attract towards the Pep-AgNPs and get killed. Therefore, the Pep-AgNPs were applied for anti-bacterial studies.

### 3.2. Pep-AgNPs Characterizations

#### 3.2.1. FT-IR Analysis

**Figure 2:**
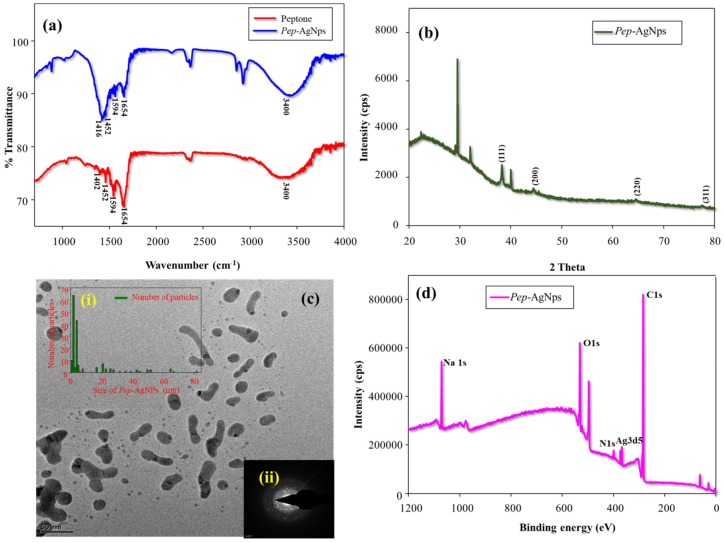
(**a**) FT-IR analysis of the peptone and Pep-AgNPs, (**b**) XRD analysis of Pep-AgNPs, (**c**) TEM images of Pep-AgNPs (i) Size histogram of Pep-AgNPs from TEM image (**c**) and (ii) SAED pattern of the Pep-AgNPs, and (**d**) XPS analysis spectrum of the Pep-AgNPs.

Surface functionalization of AgNPs with the peptone was analyzed by the FT-IR analysis. Figure 2a represents the FT-IR spectrum of peptone and Pep-AgNPs. The obtained FT-IR spectrum of peptone gave absorption peaks of 3400, 1654, 1594, and 1402 cm^−1^, which corresponds to the stretching vibration of the attached water molecule, amide I of the proteins, amide II of the proteins, and amide III of the proteins, respectively (Figure 1) [25,26]. Similar characteristic proteins peaks (amide I, amide II and amide III) also appeared in the Pep-AgNPs sample (Figure 2a). 

The obtained FT-IR results strongly corroborated a peptone functionalization of the silver nanoparticles. The proteins present over the silver nanoparticle surface may bind through the carbonyl and free amino groups, and act as a capping agent for stabilization of AgNPs [27]. 

#### 3.2.2. XRD Analysis

Crystalline purity of Pep-AgNPs was investigated by XRD analysis (Figure 2b). The obtained pattern clearly displays the characteristic silver peaks at (2θ) angle of 38.30, 44.39, 64.49, and 77.45 corresponding to the (111), (200), (220), and (311) crystalline planes, respectively. Through comparing JCPDS (file no: 89-3722), the characteristic pattern of green-synthesized AgNPs were found to possess an fcc (face-centred cubic) structure of typical AgNPs. However, the other 5 unassigned peaks obtained at (2θ) angle of 22.32, 29.08, 29.46, 32.98, and 40.05 corroborated the peptone (amino acids, peptides and protein structures) modification of AgNPs. Similarly, additional peaks appeared in the XRD profile of green-route synthesized AgNPs by *Pedalium murex* leaf extract [28].

#### 3.2.3. TEM Analysis

Structural morphology of the Pep-AgNPs was observed by TEM analysis. The TEM image showed a combination of long sheets and very small round-shaped Pep-AgNPs nano-particles. The anisotropic morphology shows different sizes and various shapes of the nanoparticle [27,28,29]. Therefore, the obtained TEM image corroborated that Pep-AgNPs has anisotropic particle morphologies. The method for obtaining the size of the distributed silver nanoparticles was used from the earlier report [17]. The size histogram of Pep-AgNPs nanoparticles was given in Figure 2c(i). The results displayed the different sizes of Pep-AgNPs. However, smaller and round-shaped nanoparticles (1–4 nm) were found to be in higher densities than the long sheets of Pep-AgNPs (40–80). A similar distribution was observed by the flavonoid-derived anisotropic silver nanoparticles [29]. The different shape and size of the Pep-AgNPs are mainly due to the peptone chemical composition. The peptone contains different amino acids, peptides, and proteins. Therefore, the obtained results confirmed the differential reduction pattern of silver by various peptone components. A similar reduction of silver by various components yielded to the anisotropic silver nano-particles were obtained by the previous reports [30,31]. Though the monodisperse nature of silver nanoparticles is the main issue associated with synthesis, the green-route synthesized anisotropic silver nanoparticles are known to have a great advantage in biological platforms, as the silver nanoparticles interact with microorganisms in a shape-dependent manner [30,32]. Therefore, the presence of different-sized particles in Pep-AgNPs might act as highly versatile anti-microbial components [32]. Figure 2c(ii) gave the SAED pattern of the Pep-AgNPs. The obtained SAED pattern confirms the crystalline purity of the Pep-AgNPs. The obtained SAED patterns are in high accordance with the obtained XRD pattern. 

#### 3.2.4. XPS Analysis

**Figure 3:**
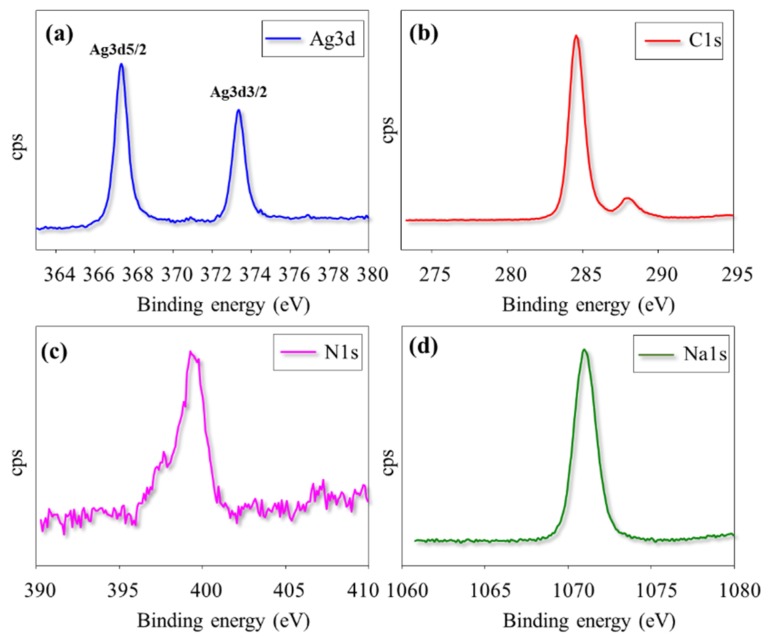
High resolution XPS spectra of (**a**) Ag3d, (**b**) C1s, (**c**) N1s and (**d**) Na1s from *Pep-AgNPs* sample.

The detailed surface elemental evaluation was made by XPS spectrum analysis. Figure 2d represents the Pep-AgNPs XPS spectrum. The obtained binding energies; 367.35, 284.6, 399.48, 1071.01, and 530.98 eV, corroborate the Ag3d5, C1s, N1s, Na1s, and O1s elemental peaks, respectively. Therefore, the obtained XPS spectrum of Pep-AgNPs confirmed successful synthesis. However, the more detailed individual high-resolution XPS spectrum was represented in Figure 3. The existence of two binding energies for Ag 3d in the Pep-AgNPs sample, 367.36, and 373.39 eV, with a difference of 6.0 eV, proved the formation of metallic silver (Figure 3a). However, the standard binding energies for pure silver are 368.1 and 374.1 eV [33]. The shift of both Ag 3d5/2 and Ag 3d3/2 to the lower binding energy in Pep-AgNPs compared to standard binding energies of the silver may be due to the occurrence of electron transfer between AgNP and peptone components. The high-resolution XPS spectrum of C1s and N1s have corroborated a typical protein structure (Figure 3b–c). Additionally, the presence of Na1s confirmed the binding of sodium salt from peptone to Pep-AgNPs. Therefore, the overall XPS investigations confirmed the successful Pep-AgNPs synthesis.

### 3.3. Anti-Bacterial Assessments of Pep-AgNPs 

**Figure 4:**
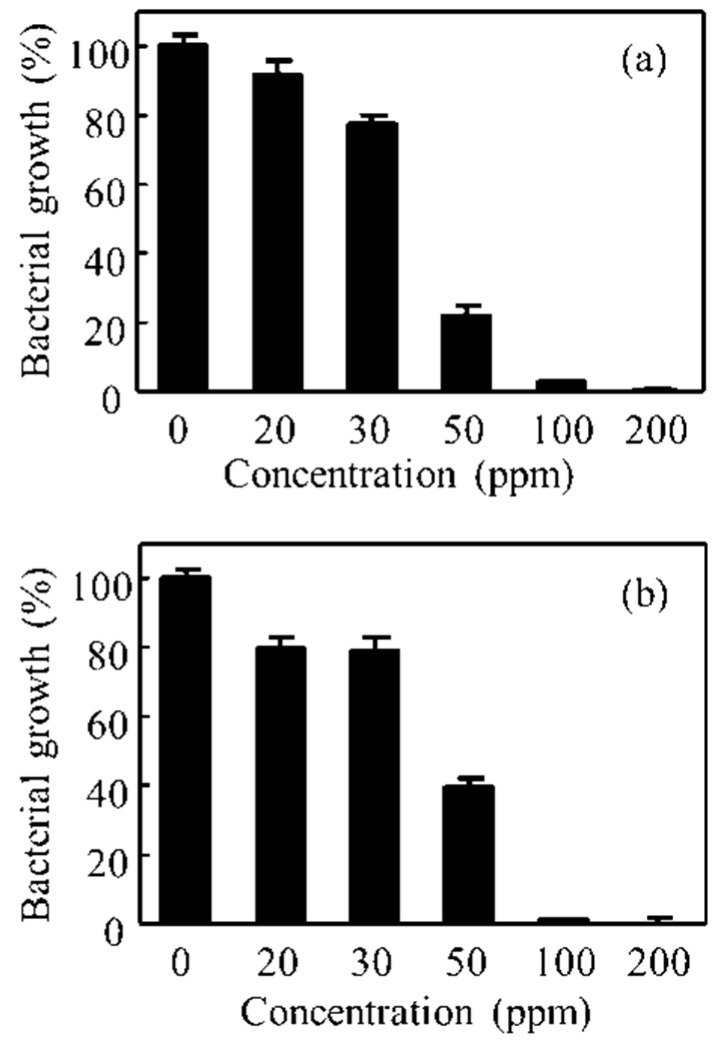
Bacterial viability of (**a**) *E. coli* and (**b**) *S. aureus*, with the various concentrations of *Pep-AgNPs*.

**Figure 5:**
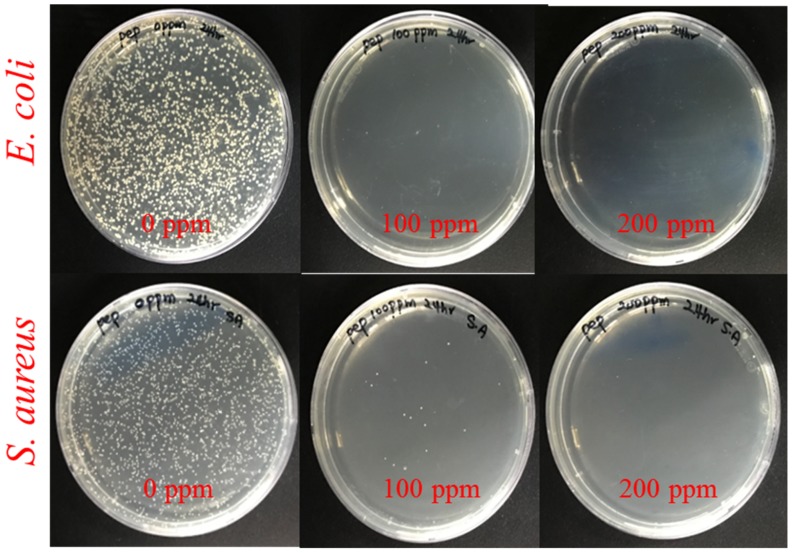
Photos showing the colonies of *E. coli* and *S. aureus* in agar petri dishes after treatment with 100 and 200 ppm of the Pep-AgNPs.

After the successful synthesis and detailed characterizations, Pep-AgNPs was applied for anti- bacterial assessment against pathogenic bacteria, such as *E. coli* and *S. aureus*. The bactericidal activity of Pep-AgNPs against the *E. coli* and *S. aureus* was investigated using MIC, CFU formation, and SEM morphology of the bacterial cells. Figure 4 shows the anti-bacterial performance of the *Pep-AgNPs* towards *E. coli* and *S. aureus* after the 4 h incubation from treatment. With the subsequent increase in the Pep-AgNPs concentration, the bacterial growth inhibition was found to be dose-dependent. Gallic acid-coated silver nanoparticles also gave the dose dependent anti-bacterial activities to both the *E. coli* and *S. aureus*, respectively [17]. Figure 4 suggests that the MIC of Pep-AgNPs was observed to be 100 ppm for *E. coli* and *S. aureus*. At the concentration of 100 ppm, the almost-complete killing of the bacteria was observed. Therefore, the MIC of 100 ppm was determined as the lowest concentration of Pep-AgNPs without further observed bacterial growth. However, the obtained results suggest that the Pep-AgNPs are potent anti-bacterial materials for the gram-positive, as well as the gram-negative, bacteria. Anisotropic silver nanoparticles of silk fibroin gave anti-bacterial activity only towards the gram-negative bacteria, and gram-positive bacteria was found to be resistant [31]. However, the anisotropic Pep-AgNPs exhibited anti-bacterial activity towards both gram-positive and gram-negative bacteria, which might be due to the bacteria getting attracted towards the nutrient peptone and increased interactions with the Pep-AgNPs. Furthermore, the CFU analysis was performed for the anti-bacterial assessment. Figure 5 shows the photos of petri dishes that have the colonies of *E. coli* and *S. aureus* against the increasing concentrations of Pep-AgNPs.

**Figure 6:**
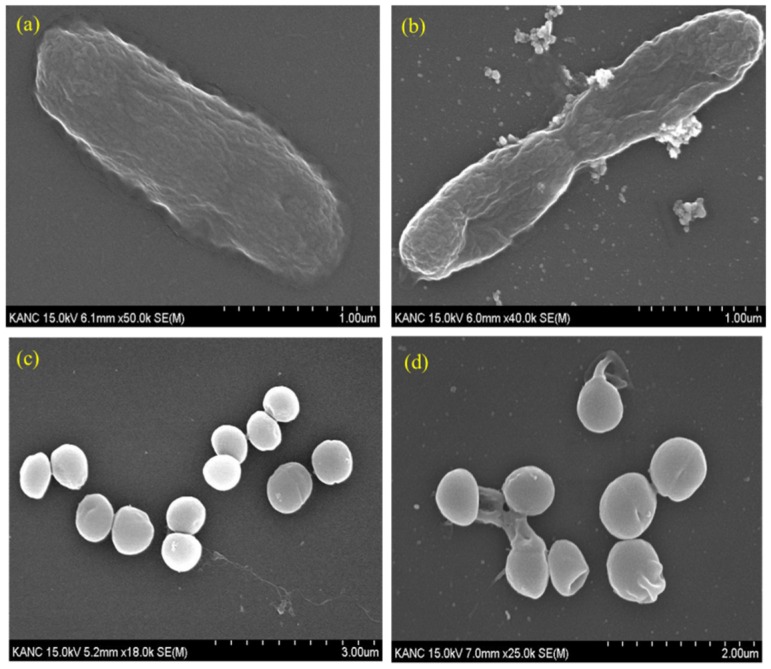
SEM image of (**a**) *E. coli* (untreated), (**b**) *E. coli* (treated with 100 ppm Pep-AgNPs), (**c**) *S. aureus* (untreated) and (**d**) *S. aureus* (treated with 100 ppm of Pep-AgNPs).

At the concentration of 100 ppm of Pep-AgNPs, no bacterial colonies were obtained in the *E-coli* culture, and very few colonies were observed in the *S. aureus* culture. The obtained results are in accordance with the earlier MIC experiment, and additionally corroborated a potent anti-bacterial capacity of the Pep-AgNPs. It is very important to observe the morphology of the bacterial cell after the silver nanoparticles treatment [14,16]. Therefore, the *E. coli* and *S. aureus* cells were observed under the SEM for analyzing the changes in morphology and membrane integrity after treatment with the Pep-AgNPs. The SEM image of the untreated *E. coli* gave an elongated rod shape (Figure 6b). The SEM image of the *E. coli* treated with Pep-AgNPs at 100 ppm revealed the shrinkage of the cell, with the formation of a porous structure and a distorted shape, from the adverse effect of Pep-AgNPs (Figure 6b). Gram-negative bacterial cell wall was made up of a thin layer of peptidoglycan, and a lipopolysaccharide layer [16]. Therefore, the Pep-AgNPs easily disrupted the membrane structure of *E. coli* and shrunk it to a distorted shape (Figure 6b). However, the gram-positive bacteria possesses thick peptidoglycan layer of linear polysaccharide chains which cross-linked with short peptides to form a unique three-dimensional structure [16]. Hence, the *S. aureus* with a rigid gram-positive cell wall did not show significant cell wall damage (Figure 6d). The leakage of the cell wall occurred at particular sites (Figure 6d) and the rest of the cell wall structure remained intact. The leakage of the intracellular material occurred at specific sites by Pep-AgNPs, which leads to the ant-bacterial effect of the *S. aureus.* Similar morphological observations after treatment with AgNPs were obtained for *E. coli* and *S. aureus* [14]. The AgNPs antibacterial activity mechanism recently stated that lipopolysaccharides (LPS) typically interact with the positively charged AgNPs, and thus cause permeability of water [17]. Specifically, AgNPs are well-known to cause more damage to the *E. coli* cell membrane and significantly lower the activity of membranous enzymes, as reported earlier [17]. 

The antibacterial mechanism of the *S. aureus* cells in the presence of AgNPs was explained by the leakage of proteins and reducing sugars from the cell [34]. Nevertheless, looking at the well-established role of peptone in bacterial growth, it can be hypothesized that micro-organisms might get attracted towards the peptone, and be easily killed by well-known anti-bacterial anisotropic silver nanoparticles. However, to elaborate on this hypothesis, further highly selective research is needed to fill the data gaps. This study aimed to reports the synthesis, process optimizations, structural characterizations, and the anti-bacterial assessment of Pep-AgNPs. Therefore, anti-bacterial silver containing microbial nutrient peptone Pep-AgNPs can be a highly efficient anti-bacterial agent for several applications.

## 4. Conclusions

In summary, we applied a well-known microbiological media component, peptone, for the synthesis of anti-bacterial AgNPs. A greener-route based high-concentration synthesis of Pep-AgNPs was successfully achieved using a substantially low concentration of peptone (0.02%). Facile-synthesized Pep-AgNPs were structurally and functionally characterized by FT-IR, XPS, XRD, and TEM analyze. TEM analyses confirmed the anisotropic nature of Pep-AgNPs. The synthetic process parameters of Pep-AgNPs were optimized for higher production of Pep-AgNPs. Furthermore, Pep-AgNPs was assessed for the subsequent anti-bacterial properties against *Escherichia coli* and *Staphylococcus aureus*. Finally, the morphological observations were made after and before the *Pep-AgNPs* treatment to study microorganisms by FE-SEM analyses. Hence, Pep-AgNPs are anisotropic, economical, can be produced without any environmentally harmful by-products, and their potent anti-bacterial agents can be applied in several anti-bacterial applications.
